# From adaptation to maladaptation: regulatory reserve and ventilatory control responses to high altitude

**DOI:** 10.3389/fphys.2026.1914164

**Published:** 2026-07-31

**Authors:** Nicolas Bourdillon, Grégoire P. Millet

**Affiliations:** Institute of Sport Sciences, University of Lausanne, Lausanne, Switzerland

**Keywords:** acclimatization, altitude, functional reserve, hypoxia, regulatory reserve

## Abstract

Altitude exposure can promote beneficial physiological adaptations but may also lead to altitude-related illnesses. Despite decades of research, the mechanisms explaining why some individuals acclimatize successfully whereas others do not remain incompletely understood. Traditional models of altitude acclimatization focus on the steps of the oxygen cascade but only partially account for the marked interindividual variability observed in acclimatization.

In this perspective, the concept of regulatory reserve, defined as the capacity of integrated physiological control systems to maintain homeostasis during environmental hypoxia is proposed. Successful acclimatization would not only depend on the functional reserves of organs at each step of the oxygen cascade, but also on the coordinated regulation of respiratory, cardiovascular, cerebrovascular, autonomic, and diffusive processes. Conversely, altitude-related illnesses may arise when this regulatory reserve becomes insufficient, exhausted, or dysregulated. Examples from ventilatory control and pulmonary diffusion are used to highlight how adaptive responses can become maladaptive when this regulatory reserve fails. The discussion focuses on how dynamic physiological markers, including breathing variability, loop gain, heart rate variability, baroreflex sensitivity, and diffusion reserve may improve the prediction of altitude acclimatization. In addition, a systems physiology approach in which acclimatization is viewed as an emergent property of interacting control networks is outlined. Such concepts may provide new insights into altitude acclimatization, maladaptation, and the prediction of human responses to altitude.

## Introduction

Altitude exposure is a physiological stressor which can be used positively in healthy ageing (Burtscher and Samaja, 2024) or to counteract the deleterious effects of some pathologies such as hypertension (Navarrete-Opazo and Mitchell, 2014), coronary disease (Burtscher et al., 2004), or chronic obstructive pulmonary disease [COPD (Haider et al., 2009)]. However, environmental hypoxia may also elicit deleterious effects in the abovementioned cases (Richalet and Lhuissier, 2015; Dong et al., 2025) and in a healthy population (Luks et al., 2017) resulting in the extensively studied acute mountain sickness [AMS (Roach et al., 2018)], high altitude cerebral oedema [HACE (Hackett and Roach, 2004)], high altitude pulmonary oedema [HAPE (Swenson and Bärtsch, 2012)], and sleep apnea (Dempsey et al., 2012; Burgess et al., 2013). Altogether, they were defined as a “conspiracy of maladaptation” (Dempsey and Morgan, 2015). Both the positive and negative effects of hypoxia are dose-dependent; e.g. determined by its severity, exposure duration and intermittent exposure pattern (Navarrete-Opazo and Mitchell, 2014; Verges et al., 2015; Burtscher et al., 2024). The literature is almost unanimous on the fact that the lack of acclimatization is the main risk factor for acute altitude illnesses (and that descent is the optimal treatment). Yet, despite decades of research, the reasons why some individuals successfully acclimatize whereas others develop altitude-related illnesses remain incompletely understood.

Over the past decades, the oxygen cascade has provided a powerful conceptual framework for understanding oxygen transport from the atmosphere to the mitochondria and the contribution of organ-specific functional reserves, such as ventilatory, cardiovascular, pulmonary diffusion, and hematological reserves, to altitude adaptation. These concepts have substantially advanced our understanding of the physiological determinants of oxygen delivery. However, they primarily describe the capacity of individual organs or transport steps and only partially explain the marked interindividual variability observed during adaptation. Indeed, individuals with apparently similar ventilatory responses, pulmonary function or cardiovascular capacities may exhibit markedly different susceptibility to altitude illness. Such observations indicate that successful acclimatization depends not only on the magnitude of physiological reserves within individual organs but also on the dynamic coordination of multiple physiological control systems across the respiratory, cardiovascular, cerebrovascular and autonomic networks.

This marked interindividual variability in adaptation has intrigued the specialists worldwide as hypoxic exposure can be both friend and foe, to this extent an entire body of literature focuses on predicting altitude related sicknesses (Richalet et al., 2012) but there are still inconsistencies and knowledge gaps. This perspective paper focuses on developing the concept of regulatory reserve, defined as the capacity of integrated physiological control systems to maintain oxygen homeostasis and physiological stability under hypoxic stress in order to better predict altitude acclimatization. Rather than replacing the classical oxygen cascade, this framework complements it by emphasizing the dynamic interactions among physiological systems that may ultimately determine successful acclimatization or maladaptation and thereby improve the prediction of altitude tolerance.

### From the oxygen cascade to an integrated control systems

Classical mechanisms of altitude adaptation rely on the oxygen cascade, which describes the sequential transport of oxygen from the ambient air to the mitochondria through ventilatory convection, pulmonary diffusion, cardiovascular convection, and peripheral (muscle) diffusion. Over the past decades, each of these components has been successfully integrated into comprehensive theoretical models that have greatly improved our understanding of oxygen transport under physiological and pathological conditions (di Prampero and Ferretti, 1990; Wagner, 1996; Bourdillon et al., 2026). The past famous expeditions to high attitude highlighted the importance of organ-specific functional reserves, such as ventilatory reserve, cardiac reserve, pulmonary diffusion reserve, or the capacity to increase hemoglobin concentration, as key determinants of tolerance to the environmental hypoxic stress (Pugh, 1976; West, 1982, 2001, 2012; Grocott et al., 2010). These reserves play a role both in the acute and chronic phases of altitude adaptation. For example, cardiac reserve is essential for the acute increase in blood flow required to compensate for the reduced arterial oxygen content. Over time, the kidneys promote a reduction in plasma volume, thereby increasing hemoglobin concentration, and sustained hypoxic exposure subsequently stimulates erythropoiesis, leading to an increase in total hemoglobin mass. As these two adaptive processes progressively develop, the relative importance of cardiac reserve declines as hemoglobin concentration and, subsequently, total hemoglobin mass increasingly contributes to oxygen transport. Altogether these reserves contribute to the broader concept of physiological reserve, namely the ability of the organism as a whole to maintain oxygen delivery and homeostasis despite reductions in environmental oxygen availability, by coordinating the contribution of each organ for global acute and chronic adaptation.

The coordinated contribution is a key element as, individuals possessing similar ventilatory capacities, cardiovascular function, or oxygen transport characteristics may exhibit markedly different acclimatization trajectories and susceptibility to altitude illness. Such observations suggest that successful adaptation is determined not only by the capacity of individual physiological systems to respond to hypoxia, but also by their ability to coordinate and regulate their responses across multiple levels of biological organization.

**Figure 1:**
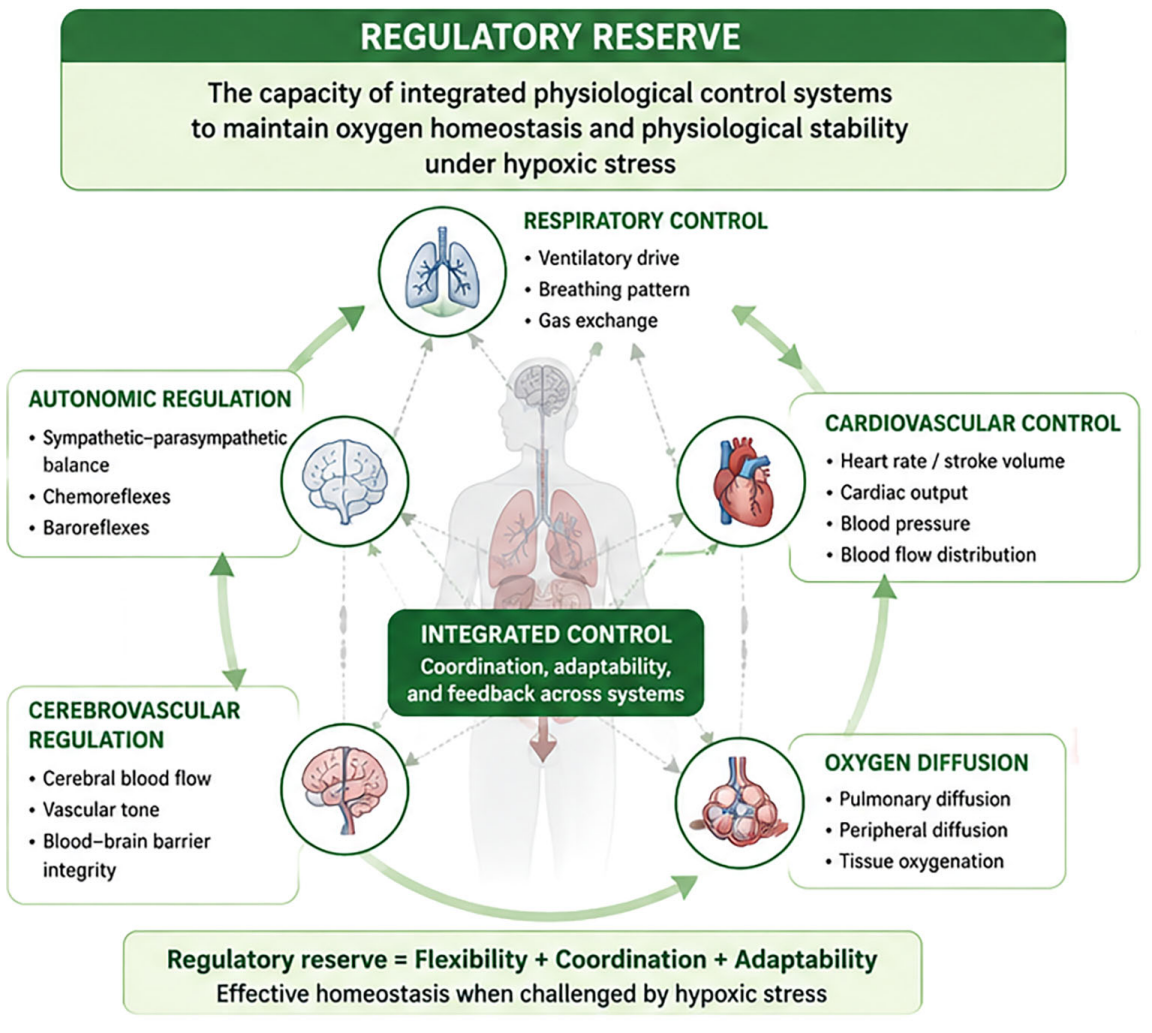
Conceptual framework illustrating the proposed interactions contributing to regulatory reserve. Solid arrows: interactions supported by current physiological evidence; dashed arrows: hypothesized interactions requiring experimental validation.

We therefore propose distinguishing physiological reserve from regulatory reserve. Whereas physiological reserve reflects the integrated functional capacity of the organism arising from the reserve of individual organs (e.g., ventilatory, cardiac, pulmonary diffusion and hematological reserves), regulatory reserve describes the capacity of the physiological control networks to coordinate the recruitment and interaction of these reserves in response to environmental hypoxia. In this framework, successful acclimatization depends not only on the magnitude of the available physiological reserves but also on the efficiency with which they are dynamically orchestrated across multiple levels of biological organization over time, as physiological regulation progressively shifts from acute compensatory responses to chronic adaptive mechanisms (**Figure 1**). Altitude illness may therefore arise not simply because one functional reserve is insufficient, but because the regulatory processes coordinating ventilation, circulation, oxygen transport, and metabolic demand fail to preserve integrated oxygen homeostasis despite substantial reserve within individual organ systems.

Consequently, the oxygen cascade, rather than a simple linear sequence extending from the atmosphere to the mitochondria, becomes a combination of interconnected feedback loops operating across multiple temporal and spatial dimensions; and the ability to regulate interactions among the various steps becomes as important as the convection/diffusion capacities themselves.

**Figure 1** summarizes the conceptual framework proposed herein, some interactions, such as those linking chemoreflex control, ventilatory instability, and cerebrovascular regulation, are supported by substantial physiological evidence, whereas others remain hypothetical or only indirectly supported. We propose that the integration of these regulatory processes, rather than their isolated behavior, deserves experimental investigation.

### Ventilatory control: is instability the key to adaptation?

In the collective unconscious, periodic breathing is almost synonymous with sleep apnea and respiratory instability. Sleep apnea is strongly associated with hypertension (Peppard et al., 2000), cardiovascular disease (Brack et al., 2012), cognitive decline (Yaffe et al., 2011) and increased mortality (Young et al., 2008). Consequently, oscillatory breathing patterns are generally perceived as pathological and undesirable as they are interpreted as evidence of an impaired control system not able to maintain adequate gas exchange. Yet, recent work demonstrated that periodic breathing in acute normobaric hypoxia reduces inspiratory effort without impairing alveolar ventilation, suggesting an adaptive mechanism to optimize respiratory efficiency in young healthy males (Heiniger et al., 2026). These latter findings reveal that periodic breathing might not only be a failure of control but may also reflect adaptation.

During wakefulness, the ins and outs of periodic breathing might be totally different as breathing is regulated not only by automatic brainstem mechanisms but also by cortical and behavioral inputs. Consequently, oscillatory breathing patterns observed in awake individuals may reflect a fundamentally different physiological phenomenon than sleep apnea. Rather than representing a failure of control, they may reveal an active regulatory process through which the respiratory system continuously adapts to changing metabolic demands and environmental constraints.

This possibility is supported by recent observations from the AltitudeOmics project which showed that breathing variability whilst awake increased substantially upon ascent to 5,260 m and remained elevated throughout the two weeks of acclimatization (Bourdillon et al., 2025). Furthermore, the individuals exhibiting greater oscillatory power at sea level or during acute hypoxic exposure developed larger increases in ventilation after acclimatization. Similar conclusions had been anticipated by other studies showing that oscillatory characteristics of breathing during wakefulness may contain predictive information regarding altitude acclimatization (Hermand et al., 2015, 2021). These observations challenge the traditional view that variability is merely noise or instability and instead suggest that breathing oscillations may contain meaningful physiological information regarding the adaptive capacity of the respiratory control system to environmental hypoxia.

The respiratory control system operates as a complex closed-loop network involving peripheral and central chemoreceptors (Xie et al., 2005, 2009; Fan et al., 2014, 2015), cerebrovascular regulation (Fan et al., 2010), pulmonary gas exchange, circulatory transport delays (Agostoni et al., 2017) and altered plant gain (Bird et al., 2023). When exposed to hypoxia, each component of this network undergoes substantial adjustments. Peripheral chemoreceptors, primarily located in the carotid bodies, respond rapidly to reductions in arterial oxygen tension and initiate the acute hypoxic ventilatory response, whereas central chemoreceptors located in the brainstem primarily sense changes in CO_2_/H^+^ concentration and shape the stability and gain of the respiratory feedback loop. Their interaction is therefore essential for ventilatory acclimatization and the emergence of breathing oscillations under hypoxic conditions. When exposed to hypoxia, each component of this network undergoes substantial adjustments. Peripheral chemosensitivity to hypoxia increases (Vizek et al., 1987), central CO_2_ responsiveness is reset (Fan et al., 2015), cerebral autoregulation is altered (Ainslie and Ogoh, 2010), and ventilatory drive progressively increases. Such modifications increase the gain of the system and seem to favor oscillatory dynamics (Bourdillon et al., 2025), which may be the sign of an adaptive system operating close to its optimal sensitivity. Thus, breathing variability could be seen as a physiological search strategy through which the respiratory controller continuously probes the hypoxic/hypocapnic environment and adjusts ventilation accordingly. Such behavior may be particularly advantageous at altitude, where oxygen and carbon dioxide equilibriums/sensitivity change drastically and the work of breathing may become a burden.

Traditionally, pulmonary reserve is defined as the excess ventilatory capacity available above resting requirements. However, the ability to tolerate environmental hypoxia depends not only on the magnitude of available ventilation but also on the flexibility of the control mechanisms governing ventilation. A subject possessing a large regulatory reserve would not only be capable of increasing ventilation when required but would also exhibit sufficient chemoreflex responsiveness, cerebrovascular regulation and autonomic coordination to dynamically adapt to environmental challenges. Breathing variability may therefore represent an observable manifestation of regulatory reserve and not only pulmonary reserve. The oscillations themselves would not be the reserve, but rather a measurable consequence of a control system capable of flexibly adjusting under challenging environmental conditions. Accordingly, recent evidence highlighted the distinction between the reserve capacity of an individual organ and the ability of the organism as a whole to respond to a physiological challenge. This concept was illustrated in a study examining sex differences in pulmonary limitations during maximal exercise (Raberin et al., 2024). Although women exhibited a lower prevalence of expiratory flow limitation than men, they experienced exercise-induced hypoxemia more frequently. These findings suggest that the greater susceptibility to hypoxemia in women reflects an integrated physiological response to the combined stress of exercise and hypoxia, rather than an intrinsic limitation in pulmonary reserve.

Paradoxically, periodic breathing/sleep apnea and adaptative variability may be the two ends of the same fundamental control architecture. The difference lies in the availability of the individual regulatory reserve. A poorly variable breathing may indicate a poorly flexible control system and could be seen as a risk factor for altitude acclimatization. In this view, oscillatory breathing is neither inherently beneficial nor pathological. Rather, it would reflect the dynamic profile of the respiratory control system and its ability—or inability—to maintain homeostasis in the face of environmental stress. Understanding where the boundary lies between adaptive variability and maladaptive periodicity may provide important insights into acclimatization and altitude-related illnesses.

Current evidence. There is substantial evidence that hypoxia modifies chemoreflex gain (Ainslie and Ogoh, 2010), cerebrovascular CO_2_ responsiveness (Fan et al., 2014), and breathing variability (Dempsey et al., 2012). Recent work also suggests that breathing variability may predict ventilatory acclimatization (Bourdillon et al., 2025). However, whether these ventilatory dynamics directly influence susceptibility to altitude illness remains unclear. We therefore propose that breathing variability represents one component of the regulatory reserve and should be investigated in conjunction with other physiological systems rather than in isolation.

### Diffusion reserve from sea level to Mount Everest

**Figure 2:**
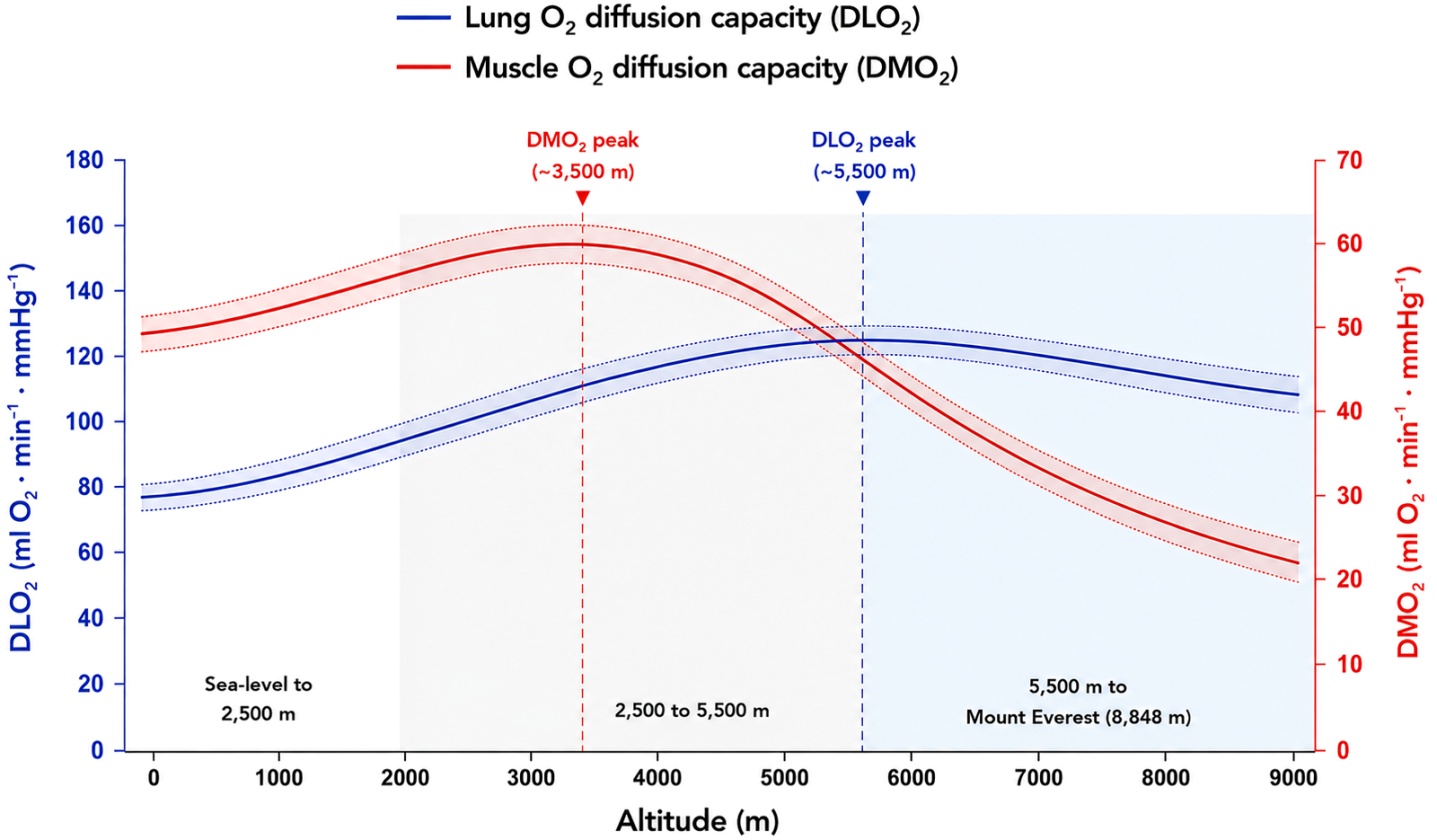
Computed lung (DLO_2_, blue line) and muscle (DMO_2_, red line) O_2_ diffusion capacity at maximal exercise intensity from sea level to the altitude of Mount Everest (8,848 m). Shaded areas and dotted lines represent the 95% confidence interval. White zone from seal-level to 2,500 m represent low altitude, grey zone from 2,500 to 5,500 represent moderate to high altitude and above 5,500 represents no permanent human life. Adapted from Bourdillon et al., 2026.

Moving one step further along the oxygen cascade, yet remaining intimately connected to ventilatory control, pulmonary oxygen diffusion provides another compelling illustration of the distinction between functional and regulatory reserves. Traditionally, pulmonary diffusion is considered a potential limiting factor for oxygen transport at altitude because the reduction in alveolar oxygen pressure decreases the driving force for oxygen diffusion from the lung alveoli to the blood (Wagner, 1996). However, a recent modeling study may indicate otherwise (Bourdillon et al., 2026). Simulations performed across altitude from sea level to mount Everest indicate that pulmonary oxygen diffusion capacity (DLO_2_) can progressively increase with hypoxic exposure and remains recruitable up to approximately 5,500 m, even at maximal exercise (**Figure 2**). These findings imply that the healthy lung possesses a substantial diffusion reserve that is largely underutilized at sea level and can be mobilized at altitude. If a significant pulmonary diffusion reserve remains available, then many altitude-related disorders occurring below approximately 5,500 m cannot be explained solely by an intrinsic limitation of the lung capacity for oxygen diffusion. Rather, they may originate from disturbances in the mechanisms regulating ventilation.

High-altitude pulmonary oedema (HAPE) provides a striking example of this distinction. HAPE does not arise because the lung has exhausted its diffusion capacity. Instead, it results from a maladaptive response of the pulmonary circulation to environmental hypoxia. Hypoxic pulmonary vasoconstriction is initially a response to environmental hypoxia aiming at improving the ventilation/perfusion ratio. However, excessive hypoxic pulmonary vasoconstriction increases pulmonary arterial pressure, and ultimately leads to capillary stress failure and fluid accumulation in the lung alveoli (West et al., 1991). Ironically, this dysregulation of pulmonary vascular control subsequently creates the very diffusion limitation that was not initially present. The pathological reduction in gas exchange is therefore a consequence rather than a cause of the maladaptive response.

Pulmonary diffusion reserve can be viewed as a functional reserve, whereas the coordinated control of ventilation and pulmonary vascular function represents a component of regulatory reserve; and successful acclimatization requires both. A large diffusion reserve may provide the potential for maintaining/limiting the decrease in oxygen diffusion under environmental hypoxia, but this potential can only be realized if the regulatory systems governing ventilation and pulmonary perfusion remain appropriately coordinated. Conversely, maladaptation may emerge despite substantial structural reserve when regulatory mechanisms fail. Therefore, tolerance to altitude is determined both by the capacity of the organs involved (presently the lungs), but also by the ability of the control systems to orchestrate their responses to the environmental stress.

Current evidence. Pulmonary diffusion reserve (Wagner, 2015) and hypoxic pulmonary vasoconstriction (Sylvester et al., 2012) are individually well characterized. Likewise, excessive hypoxic pulmonary vasoconstriction is an established mechanism underlying HAPE (Swenson and Bärtsch, 2012). However, whether the interaction between ventilatory control, pulmonary vascular regulation, and diffusion reserve can predict individual susceptibility to HAPE has not been systematically investigated yet. We propose that this interaction represents one expression of regulatory reserve.

### Predicting altitude tolerance

One of the challenges in altitude physiology is the remarkable interindividual variability in acclimatization. Despite exposure to the same environmental stressor, some individuals rapidly adapt and remain symptom-free, whereas others develop substantial physiological impairment or altitude-related illness. Although numerous predictors have been proposed (including oxygen saturation, hypoxic ventilatory response, exercise capacity, and previous altitude-related illnesses history) and the Richalet test (Richalet et al., 2012) was designed for altitude illness prediction, the latter remains incomplete. The central question therefore remains: can maladaptation to altitude be better predicted?

In facts the improvement might just be around the corner, relying on the existing test/measurement the already determined markers but analyzing the data slightly differently. For example, adding the ventilatory variability analysis to the Richalet test (Hermand et al., 2015, 2021) might help make prediction more accurate and thinking in terms of regulatory reserve rather than amplitude of changes in physiological parameters might also conceptually help the prediction. In this view, altitude acclimatization would be determined by the collective adaptability of the respiratory, cardiovascular, cerebrovascular, and pulmonary systems. Individuals with greater regulatory reserve would be expected to accommodate hypoxic perturbations more effectively and therefore acclimatize more successfully.

For example, at present there is no direct evidence demonstrating that sleep periodic breathing contributes to HAPE development. Both phenomena arise from distinct physiological mechanisms, ventilatory instability in one case and excessive pulmonary vascular responses in the other. However, both are influenced by chemoreflex sensitivity, pulmonary gas exchange, and the overall regulation of oxygen homeostasis. We therefore hypothesize that they may represent different manifestations of a common limitation in regulatory reserve, a possibility that remains to be experimentally tested.

Future approaches to altitude prediction may therefore benefit from moving beyond the classical measurement interpretations and toward dynamic biomarkers of physiological regulation. Additional markers should include the capture of the flexibility of ventilatory control; loop gain, and cerebral autoregulation, which quantify the stability of the respiratory feedback system and sensitivity to oxygen and carbon dioxide; heart rate variability, reflecting autonomic adaptability; baroreflex sensitivity, indicating cardiovascular control reserve; and estimates of pulmonary diffusion reserve (Bourdillon and Millet, 2026), which may characterize the capacity of the lung to maintain gas exchange during environmental hypoxic stress. The predictive value of these biomarkers may reside less in their absolute values than in their ability to reveal system flexibility and responsiveness. This distinction may help explain why traditional markers sometimes fail to predict altitude outcomes consistently across populations.

Consequently, regulatory reserve can be viewed as a property of the integrated physiological network rather than of any individual component. Reserve exists not only in the capacity of each subsystem to respond to perturbations but also in the flexibility of their interactions. A decline in one component may be compensated for by adjustments elsewhere within the network, whereas simultaneous limitations across multiple systems may precipitate maladaptation. In fact, the people for whom the current altitude acclimatization prediction fails may be the ones for whom individual component of the oxygen cascade respond favorable, but not their interconnected network. For these individuals, the emergence of altitude illness may therefore reflect a breakdown in network coordination rather than the failure of a single physiological process. Regulatory reserve is not directly measurable but may be inferred from the coordinated behavior of dynamic physiological markers, including breathing variability, ventilatory loop gain, heart rate variability, baroreflex sensitivity, cerebrovascular regulation and pulmonary diffusion reserve.

This perspective focuses specifically on the regulation of acute altitude acclimatization and the transition toward maladaptation during the first hours to weeks of lowlanders’ exposure to altitude. Long-term evolutionary adaptations observed in high-altitude populations, such as Tibetans and Andeans, involve additional genetic and developmental mechanisms that fall beyond the scope of the present article.

## Conclusion

The physiological and pathological responses to high altitude may not represent distinct phenomena but rather different positions along a continuum of regulatory reserve. Understanding how ventilatory control, oxygen diffusion, and autonomic regulation interact may provide a complementary helpful way to predicting altitude acclimatization, identifying maladaptation, and developing personalized strategies for altitude exposure.

## Data Availability

The original contributions presented in the study are included in the article. Further inquiries can be directed to the corresponding author.

## References

[B1] Agostoni P. Corrà U. Emdin M. (2017). Periodic breathing during incremental exercise. Ann. Am. Thorac. Soc 14, S116–S122. doi: 10.1513/AnnalsATS.201701-003FR 28443680

[B2] Ainslie P. N. Ogoh S. (2010). Regulation of cerebral blood flow in mammals during chronic hypoxia: a matter of balance. Exp. Physiol. 95, 251–262. doi: 10.1113/expphysiol.2008.045575 19617269

[B3] Bird J. D. Sands S. A. Alex R. M. Shing C. L. H. Shafer B. M. Jendzjowsky N. G. . (2023). Sex-related differences in loop gain during high-altitude sleep-disordered breathing. Ann. Am. Thorac. Soc 20, 1192–1200. doi: 10.1513/AnnalsATS.202211-918OC 37000675 PMC10405604

[B4] Bourdillon N. Fan J.-L. Elliott J. E. Millet G. P. Subudhi A. W. Kayser B. . (2025). AltitudeOmics: breathing variability at rest and during exercise across 16 days of acclimatization to hypobaric hypoxia. J. Physiol. doi: 10.1113/JP289619 41400500

[B5] Bourdillon N. Manferdelli G. Raberin A. Millet G. P. (2026). Modelling lung and muscle oxygen diffusion capacities from sea-level to Mount Everest. Sci. Rep. 16, 7817. doi: 10.1038/s41598-025-32441-9 41771934 PMC12953604

[B6] Bourdillon N. Millet G. P. (2026). Taking heart rate variability to the next level in sports: towards a multi-signal integration. Front. Sports. Act. Living. 8, 1720495. doi: 10.3389/fspor.2026.1720495 41657468 PMC12872898

[B7] Brack T. Randerath W. Bloch K. E. (2012). Cheyne-Stokes respiration in patients with heart failure: prevalence, causes, consequences and treatments. Respir. Int. Rev. Thorac. Dis. 83, 165–176. doi: 10.1159/000331457 22025128

[B8] Burgess K. R. Lucas S. J. E. Shepherd K. Dawson A. Swart M. Thomas K. N. . (2013). Worsening of central sleep apnea at high altitude--a role for cerebrovascular function. J. Appl. Physiol. 114, 1021–1028. doi: 10.1152/japplphysiol.01462.2012 23429871

[B9] Burtscher J. Citherlet T. Camacho‐Cardenosa A. Camacho‐Cardenosa M. Raberin A. Krumm B. . (2024). Mechanisms underlying the health benefits of intermittent hypoxia conditioning. J. Physiol. 602, 5757–5783. doi: 10.1113/JP285230 37860950

[B11] Burtscher M. Pachinger O. Ehrenbourg I. Mitterbauer G. Faulhaber M. Pühringer R. . (2004). Intermittent hypoxia increases exercise tolerance in elderly men with and without coronary artery disease. Int. J. Cardiol. 96, 247–254. doi: 10.1016/j.ijcard.2003.07.021 15262041

[B10] Burtscher J. Samaja M. (2024). Healthy aging at moderate altitudes: hypoxia and hormesis. Gerontology 70, 1152–1160. doi: 10.1159/000541216 39348814

[B12] Dempsey J. A. Morgan B. J. (2015). Humans in hypoxia: a conspiracy of maladaptation?! Physiology 30, 304–316. doi: 10.1152/physiol.00007.2015 26136544 PMC4491526

[B13] Dempsey J. A. Smith C. A. Blain G. M. Xie A. Gong Y. Teodorescu M. (2012). Role of central/peripheral chemoreceptors and their interdependence in the pathophysiology of sleep apnea. Adv. Exp. Med. Biol. 758, 343–349. doi: 10.1007/978-94-007-4584-1_46 23080181

[B14] di Prampero P. E. Ferretti G. (1990). Factors limiting maximal oxygen consumption in humans. Respir. Physiol. 80, 113–127. doi: 10.1016/0034-5687(90)90075-a 2218094

[B15] Dong Y. Wang Q. Wang X. Liu C. Hussain I. Ma H. . (2025). High altitude might increase risk of incident frailty in older adults: a nationwide longitudinal survey. BMC Public Health 25, 2513. doi: 10.1186/s12889-025-23713-0 40684121 PMC12275372

[B16] Fan J.-L. Burgess K. R. Thomas K. N. Peebles K. C. Lucas S. J. E. Lucas R. A. I. . (2010). Influence of indomethacin on ventilatory and cerebrovascular responsiveness to CO2 and breathing stability: the influence of PCO2 gradients. Am. J. Physiol. Regul. Integr. Comp. Physiol. 298, R1648–R1658. doi: 10.1152/ajpregu.00721.2009 20042691

[B17] Fan J.-L. Subudhi A. W. Duffin J. Lovering A. T. Roach R. C. Kayser B. (2015). AltitudeOmics: resetting of cerebrovascular CO2 reactivity following acclimatization to high altitude. Front. Physiol. 6, 394. doi: 10.3389/fphys.2015.00394 26779030 PMC4705915

[B18] Fan J.-L. Subudhi A. W. Evero O. Bourdillon N. Kayser B. Lovering A. T. . (2014). AltitudeOmics: enhanced cerebrovascular reactivity and ventilatory response to CO2 with high-altitude acclimatization and reexposure. J. Appl. Physiol. Bethesda. Md. 1985 116, 911–918. doi: 10.1152/japplphysiol.00704.2013 24356520

[B19] Grocott M. P. W. Martin D. S. Wilson M. H. Mitchell K. Dhillon S. Mythen M. G. . (2010). Caudwell xtreme everest expedition. High Alt. Med. Biol. 11, 133–137. doi: 10.1089/ham.2009.1093 20586597

[B20] Hackett P. H. Roach R. C. (2004). High altitude cerebral edema. High Alt. Med. Biol. 5, 136–146. doi: 10.1089/1527029041352054 15265335

[B21] Haider T. Casucci G. Linser T. Faulhaber M. Gatterer H. Ott G. . (2009). Interval hypoxic training improves autonomic cardiovascular and respiratory control in patients with mild chronic obstructive pulmonary disease. J. Hypertens. 27, 1648–1654. doi: 10.1097/HJH.0b013e32832c0018 19387363

[B22] Heiniger G. Piquilloud L. Solelhac G. Imler T. Waeber A. Bradley B. . (2026). Altitude-induced periodic breathing optimises respiratory efficiency during sleep in young healthy males. Thorax, thorax-2025-224436. doi: 10.1136/thorax-2025-224436 41980820

[B23] Hermand E. Lhuissier F. J. Pichon A. Voituron N. Richalet J.-P. (2021). Exercising in hypoxia and other stimuli: heart rate variability and ventilatory oscillations. Life. Bsl. Switz. 11, 625. doi: 10.3390/life11070625 34203350 PMC8306822

[B24] Hermand E. Pichon A. Lhuissier F. J. Richalet J.-P. (2015). Periodic breathing in healthy humans at exercise in hypoxia. J. Appl. Physiol. Bethesda. Md. 1985 118, 115–123. doi: 10.1152/japplphysiol.00832.2014 25554800

[B25] Luks A. M. Swenson E. R. Bärtsch P. (2017). Acute high-altitude sickness. Eur. Respir. Rev. 26, 160096. doi: 10.1183/16000617.0096-2016 28143879 PMC9488514

[B26] Navarrete-Opazo A. Mitchell G. S. (2014). Therapeutic potential of intermittent hypoxia: a matter of dose. Am. J. Physiol. Regul. Integr. Comp. Physiol. 307, R1181–R1197. doi: 10.1152/ajpregu.00208.2014 25231353 PMC4315448

[B27] Peppard P. E. Young T. Palta M. Skatrud J. (2000). Prospective study of the association between sleep-disordered breathing and hypertension. N. Engl. J. Med. 342, 1378–1384. doi: 10.1056/NEJM200005113421901 10805822

[B28] Pugh L. G. (1976). Physiology on mount everest, (1953) and on the preparatory expedition to Mount Cho Oyu, (1952): a demonstration of equipment and results [proceedings. J. Physiol. 263, 95P–97P. 796447

[B29] Raberin A. Manferdelli G. Schorderet F. Bourdillon N. Millet G. P. (2024). Fitness level- and sex-related differences in pulmonary limitations to maximal exercise in normoxia and hypoxia. Med. Sci. Sports. Exerc. 56, 1398–1407. doi: 10.1249/MSS.0000000000003433 38530208 PMC11882191

[B30] Richalet J.-P. Larmignat P. Poitrine E. Letournel M. Canouï-Poitrine F. (2012). Physiological risk factors for severe high-altitude illness: a prospective cohort study. Am. J. Respir. Crit. Care Med. 185, 192–198. doi: 10.1164/rccm.201108-1396OC 22071330

[B31] Richalet J.-P. Lhuissier F. J. (2015). Aging, tolerance to high altitude, and cardiorespiratory response to hypoxia. High Alt. Med. Biol. 16, 117–124. doi: 10.1089/ham.2015.0030 25946570

[B32] Roach R. C. Hackett P. H. Oelz O. Bärtsch P. Luks A. M. MacInnis M. J. . (2018). The 2018 lake louise acute mountain sickness score. High Alt. Med. Biol. 19, 4–6. doi: 10.1089/ham.2017.0164 29583031 PMC6191821

[B33] Swenson E. R. Bärtsch P. (2012). “ High‐altitude pulmonary edema,” in Comprehensive Physiology. Ed. Prakash Y. S. (Hoboken, NJ, USA: Wiley), 2753–2773. doi: 10.1002/cphy.c100029 23720264

[B34] Sylvester J. T. Shimoda L. A. Aaronson P. I. Ward J. P. T. (2012). Hypoxic pulmonary vasoconstriction. Physiol. Rev. 92, 367–520. doi: 10.1152/physrev.00041.2010 22298659 PMC9469196

[B35] Verges S. Chacaroun S. Godin-Ribuot D. Baillieul S. (2015). Hypoxic conditioning as a new therapeutic modality. Front. Pediatr. 3. doi: 10.3389/fped.2015.00058 26157787 PMC4476260

[B36] Vizek M. Pickett C. K. Weil J. V. (1987). Increased carotid body hypoxic sensitivity during acclimatization to hypobaric hypoxia. J. Appl. Physiol. 63, 2403–2410. doi: 10.1152/jappl.1987.63.6.2403 3436874

[B37] Wagner P. D. (1996). A theoretical analysis of factors determining VO2 MAX at sea level and altitude. Respir. Physiol. 106, 329–343. doi: 10.1016/s0034-5687(96)00086-2 9017851

[B38] Wagner P. D. (2015). The physiological basis of pulmonary gas exchange: implications for clinical interpretation of arterial blood gases. Eur. Respir. J. 45, 227–243. doi: 10.1183/09031936.00039214 25323225

[B39] West J. B. (1982). American medical research expedition, to everest 1981. Physiologist 25, 36–38. doi: 10.1055/s-2007-1011441 7079304

[B40] West J. B. (2001). Letter from chowri kang. High Alt. Med. Biol. 2, 311–313. doi: 10.1089/152702901750265422 11443013

[B41] West J. B. (2012). Centenary of the Anglo‐American high‐altitude expedition to Pikes Peak. Exp. Physiol. 97, 1–9. doi: 10.1113/expphysiol.2011.058776 21948193

[B42] West J. B. Tsukimoto K. Mathieu-Costello O. Prediletto R. (1991). Stress failure in pulmonary capillaries. J. Appl. Physiol. 70, 1731–1742. doi: 10.1152/jappl.1991.70.4.1731 2055852

[B43] Xie A. Skatrud J. B. Barczi S. R. Reichmuth K. Morgan B. J. Mont S. . (2009). Influence of cerebral blood flow on breathing stability. J. Appl. Physiol. Bethesda. Md. 1985 106, 850–856. doi: 10.1152/japplphysiol.90914.2008 19118158 PMC2660251

[B44] Xie A. Skatrud J. B. Khayat R. Dempsey J. A. Morgan B. Russell D. (2005). Cerebrovascular response to carbon dioxide in patients with congestive heart failure. Am. J. Respir. Crit. Care Med. 172, 371–378. doi: 10.1164/rccm.200406-807OC 15901613

[B45] Yaffe K. Laffan A. M. Harrison S. L. Redline S. Spira A. P. Ensrud K. E. . (2011). Sleep-disordered breathing, hypoxia, and risk of mild cognitive impairment and dementia in older women. JAMA 306 (6), 613–619. doi: 10.1001/jama.2011.1115 21828324 PMC3600944

[B46] Young T. Finn L. Peppard P. E. Szklo-Coxe M. Austin D. Nieto F. J. . (2008). Sleep disordered breathing and mortality: eighteen-year follow-up of the Wisconsin sleep cohort. Sleep 31, 1071–1078. doi: 10.1016/0753-3322(93)90109-x 18714778 PMC2542952

